# Prophylactic Vitamin D Prescribing for Adult Inpatients on Psychiatric Wards at Warneford Hospital – an Audit

**DOI:** 10.1192/bjo.2025.10596

**Published:** 2025-06-20

**Authors:** Justyna Gromala, Sagar Jobanputra

**Affiliations:** 1 Oxford University Hospitals NHS Trust, Oxford, United Kingdom; 2 Oxford Health NHS Trust, Oxford, United Kingdom

## Abstract

**Aims:** Vitamin D deficiency is a common and preventable problem seen in the UK, which can contribute to physical and mental health illness. The Department of Health and Social Care states that adults in restricted settings, such as inpatient psychiatric units, are at increased risk of vitamin D deficiency due to limited exposure to sunlight. Local Trust guidelines specify that all adult inpatients should be offered vitamin D prophylaxis year-round, unless there are symptoms of osteomalacia, in which case vitamin D serum levels should be tested and a treatment dose regime prescribed. Despite this, we noted that many patients did not receive prophylactic treatment. The primary aim of this audit was to increase the number of prophylactic vitamin D prescriptions for psychiatric inpatients at Warneford Hospital, in line with local Trust guidelines and national guidance.

**Methods:** We firstly collected data on current prescribing of prophylactic vitamin D for adult inpatients across 3 wards. We then re-formatted local Trust guidelines on vitamin D prescribing and testing into a simplified poster and flow chart. Copies were distributed to doctor’s offices in three adult inpatient wards, 2 female and 1 male, at the Warneford Hospital. We then compared the number of patients who were prescribed prophylactic vitamin D pre- and post-intervention.

**Results:** There were 51 patients across all 3 wards, 33 female and 18 male. In a two-week timeframe, the total number of patients who were prescribed prophylactic vitamin D doubled, from 8 (16%) to 16 (31%) patients. The biggest increase was seen in patients on the male ward, with a 5-fold increase from 1 (6%) to 5 (28%) patients. The patients on the ward changed in this timeframe, due to the normal flow of discharges and admissions on acute wards.

**Conclusion:** There was an increase in the numbers of patients who had prophylactic vitamin D supplementation prescribed in all included inpatient wards, showing that presenting guidelines in a simplified manner is a useful intervention to improve prescribing practices. Our intervention could be further improved by using additional channels of communication, such as e-mails or presentations for prescribers. A qualitative approach may be useful to explore existing barriers for patients to accept vitamin D prescriptions. In future, reducing the number of serum vitamin D level tests due to prophylactic prescribing of vitamin D in the absence of osteomalacia could have significant financial and environmental benefits for the Trust and the NHS.

